# Switches of SOX17 and SOX2 expression in the development of squamous metaplasia and squamous intraepithelial lesions of the uterine cervix

**DOI:** 10.1002/cam4.3201

**Published:** 2020-07-09

**Authors:** Jobran M. Moshi, Klaas J. Hoogduin, Monique Ummelen, Mieke E. R. Henfling, Manon van Engeland, Kim A. D. Wouters, Hans Stoop, Imke Demers, Leendert H. J. Looijenga, Frans C. S. Ramaekers, Anton N. H. Hopman

**Affiliations:** ^1^ Department of Molecular Cell Biology GROW School for Oncology & Developmental Biology Maastricht University Medical Centre Maastricht The Netherlands; ^2^ Department of Medical Laboratory Technology Faculty of Applied Medical Sciences Jazan University Jazan Kingdom of Saudi Arabia; ^3^ Laboratory of Pathology Pathan B.V. Rotterdam The Netherlands; ^4^ Department of Pathology GROW School for Oncology & Developmental Biology Maastricht University Medical Centre Maastricht The Netherlands; ^5^ Laboratory for Experimental Patho‐Oncology Department of Pathology Erasmus University Medical Centre Rotterdam The Netherlands; ^6^ Princess Máxima Center for Pediatric Oncology Utrecht The Netherlands

**Keywords:** cervical preneoplasia, keratins, reserve cells, SOX17, SOX2, squamocolumnar junction, squamous intraepithelial lesions, transformation zone

## Abstract

**Aims:**

The dynamics and topographical distribution of SOX17 and SOX2 expression was studied in the transformation zone (TZ) of the uterine cervix. This TZ is a dynamic area where switches from glandular into squamous epithelium can be recognized, new squamocolumnar junctions are formed, and premalignant lesions originate. SOX17 and SOX2 show mutually exclusive expression patterns in the normal uterine cervix, with SOX2 being exclusively found in squamous epithelium, while SOX17 is detected in endocervical columnar cells and reserve cells.

**Methods and Results:**

Normal cervices and squamous intraepithelial lesions (SIL) were studied with immunohistochemistry, methylation of SOX17, human papilloma virus (HPV) genotyping, and in situ hybridization. In the TZ squamous metaplasia originating from these reserve cells can still show SOX17 expression, while also remnants of SOX17‐positive immature metaplasia can be recognized in the normal squamous epithelium. SOX17 expression is gradually lost during maturation, resulting in the exclusive expression of SOX2 in the majority of (SIL). This loss of SOX17 expression is independent of methylation of the CpG island in its promotor region. HPV can be detected in SOX17‐positive immature metaplastic regions in the immediate vicinity of SOX2‐positive SIL, suggesting that switches in SOX17 and 2 expression can occur upon HPV infection.

**Conclusions:**

This switch in expression, and the strong association between the distribution of reserve cells and squamous areas within the columnar epithelium in the TZ, suggests that reserve cell proliferations, next to basal cells in the squamous epithelium, are potential targets for the formation of squamous lesions upon viral infection.

## Graphical abstract

Switches of SOX17 and SOX2 expression in the development of squamous metaplasia and squamous intraepithelial lesions of the uterine cervix are associated with viral infection.

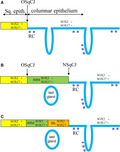

## 1 INTRODUCTION

The premalignant lesions of the uterine cervix originate in the transformation zone (TZ), an area where endocervical columnar cells are replaced by squamous epithelium. These two epithelial tissue types merge at the squamocolumnar junction (SqCJ). ^1^, ^2^, ^3^, ^4^, ^5^, ^6^ The process of epithelial replacement, taking place proximal to this mucosal junction, is called squamous metaplasia. It can be observed before birth and is particularly prominent during adolescence.^6^, ^7^, ^8^, ^9^ Squamous metaplasia is the result of proliferation and maturation of a unique subset of progenitor cells, the so‐called reserve cells, which are sporadically seen under endocervical columnar cells up to the second junction at the beginning of the endometrium.^1^, ^7^, ^10^ Upon differentiation, reserve cells acquire squamous characteristics, initially forming immature squamous metaplastic epithelium, subsequently maturating into a squamous epithelium that is indistinguishable from the original ectocervical squamous epithelium.^6^

There is a general consensus that reserve cells can be regarded as a progenitor cell population, capable of generating squamous type epithelium.^6^, ^11^, ^12^, ^13^ Several models have been proposed to describe the origin of reserve cells and their involvement in the formation of premalignant lesions.^6^, ^14^, ^15^, ^16^ Both squamous and glandular‐type carcinomas develop in the cervical TZ, which is an important argument for the hypothesis that the reserve cell plays a central role in the pathogenesis of these malignancies.^11^, ^12^, ^13^, ^17^, ^18^, ^19^ It is widely speculated that as a result of persistent human papilloma virus (HPV) infection in the TZ, the normal proliferation and differentiation of reserve cells may be derailed and a precursor lesion for these cervical carcinomas develops.^20^, ^21^ It has been demonstrated that atypical reserve cell proliferations harbor oncogenic HPV types, thereby confirming early studies, which were based on morphologic observations, sometimes in combination with biomarkers.^17^, ^22^, ^23^ These also provided evidence that the reserve cell is the progenitor for cervical carcinomas and their precursor conditions.

On the basis of marker expression patterns, particularly of keratin 7, this theory has been challenged by Herfs et al^15^ These authors identified a small population of keratin 7‐positive cuboidal cells in the vicinity of the SqCJ, which were speculated to be the progenitors of high‐grade squamous intraepithelial lesions (HSIL). Furthermore, these cuboidal cells were suggested to undergo reverse differentiation into (basal) reserve cells, which in turn can undergo metaplastic changes. However, in earlier studies,^7^, ^16^, ^24^ in which keratin phenotypes of the uterine cervix were analyzed both during embryonal development and malignant transformation, we found extensive expression of keratin 7 in all endocervical epithelial cells. On the basis of these results, and additional studies with stem cell markers,^8^, ^17^ it was concluded that reserve cells are potential progenitors of both squamous‐ and glandular cervical lesions.

In the underlying study, we examined the role of the cell lineage markers SOX17 and SOX2, transcription regulator proteins playing a pivotal role in human development.^25^, ^26^ These markers have been studied so far only separately during the development of cervical cancer. It was suggested previously that SOX2 expression increased with severity of SIL lesions, but the data in literature are contradictory.^27^, ^28^, ^29^, ^30^, ^31^, ^32^, ^33^ The expression of SOX17, on the other hand, decreases with severity of the lesion and the majority of squamous and glandular (pre)malignant lesions of the uterine cervix have been described to show methylation of the promoter CpG island region of the *SOX17* gene.^34^, ^35^

Recently, we showed that SOX17 and SOX2 exhibit a mutually exclusive expression pattern in the normal cervical epithelium, with SOX17 exclusively being found in the glandular epithelium and SOX2 exclusively expressed in the squamous epithelium. A sharp delineation of these two expression patterns was seen at the old and new SqCJs (NSqCJs).^35^ Interestingly, the reserve cells underlying the glandular epithelium were found to be SOX17 positive. When studying the topographical distribution of these two markers in squamous and glandular preneoplastic lesions (SIL and adenocarcinoma in situ; AIS), we noted an almost exclusive expression of SOX2 in SIL and a more complex, combined SOX2 and SOX17 expression pattern in AIS. Furthermore, we could show an unexpected downregulation of SOX17 in a fraction of AIS lesions, which could be explained by the methylation status of its gene promotor region.

Triggered by these earlier, more static findings we now wanted to study the dynamics of SOX2 and SOX17 expression in the carcinogenesis of the uterine cervix, with a focus on the origin of the very early stages of squamous preneoplasia and the role of the reserve cells in this process. As described above, in this respect, the TZ is an important area where switches from glandular into squamous epithelium can be recognized, NSqCJs are formed within the columnar epithelium^6^ and premalignant lesions originate.^35^ It is in such regions that we examined the relationship between the expression of the SOX17 and SOX2 transcription regulatory proteins in normal, hyperplastic, metaplastic, and premalignant epithelium. In addition, the methylation status of *SOX17* was examined to explain the observed loss of expression of the protein during the development of metaplasia. The presence of reserve cell differentiation markers (ie, keratins 17 and 7), markers for immature metaplasia (keratin 17 positivity and p16 negativity), as well as HPV type and physical status of the virus are also assessed.

Based on our findings, we provide new insights into the involvement of reserve cells and the metaplastic epithelium in the formation of preneoplastic squamous lesions in the TZ of the uterine cervix.

## 2 MATERIALS AND METHODS

### 2.1 Tissues

The following types of tissue samples were selected from the archives of the Departments of Pathology of the Foundation of Collaborating Hospitals in Eastern Groningen, Pathan Rotterdam, the Reinier de Graaf Hospital Delft, and the Maastricht University Medical Center, Maastricht, The Netherlands:

1. Formalin‐fixed and paraffin‐embedded (FFPE) tissues from 12 normal cervices removed for non‐cervix‐related conditions during hysterectomy of premenopausal women. In 11 of the 12 samples, reserve cells could be detected. There was no previous history of cervical abnormalities. To this end, patient history with regards to previous cervix smears, use of hormonal therapy, and any other relevant pathology was reviewed.
2. Formalin‐fixed and paraffin‐embedded tissues from representative samples of cervical squamous (pre)neoplastic lesions were selected, including 17 cases of low‐grade squamous intraepithelial lesions (LSIL), 33 cases of HSIL, and 20 cases of squamous cell carcinoma (SCC), as well as 10 SIL cases with co‐existing AIS, of which two were LSIL and eight were HSIL. Within these tissue samples, we analysed 27 morphologically normal areas of squamous epithelium, including remnants of (immature) metaplasia distant from the lesions, as well 13 normal areas adjacent or in close proximity to the lesion. Sections were re‐evaluated by two pathologists who selected representative tissue blocks in each case. Regions with microglandular hyperplasia were occasionally found in these samples. In all these cases, SOX17 and SOX2 expression was assessed by immunohistochemistry.

From this series of eight LSILs and 10 HSILs, as well as five cases of co‐existing SIL/AIS lesions were tested for methylation of *SOX17*. This series was also analysed for HPV by in situ hybridization (ISH) and immunostained for p16 and the differentiation markers keratin 7 and 17 in individual cases (see below).

Research on these tissue samples has been performed in accordance with the Code for Proper Secondary Use of Human Tissue in The Netherlands (http://www.federa.org/, update 2011) and has been approved by the Medical Ethical Committees of the Erasmus University Medical Centre, Rotterdam (registration numbers MEC 02‐981 and CCR2041) and of the Foundation of Collaborating Hospitals of Eastern Groningen, the Netherlands.

### 2.2 Immunohistochemistry

Immunohistochemical staining on 4‐µm thick FFPE tissue sections was performed using primary antibodies against SOX17, SOX2, keratin 7, keratin 17, and p16. Detailed information on antibodies and immunostaining conditions is summarized in the Materials and Methods S1, and in Table S1. The sections were scanned with a Ventana iScan HT slide scanner (Ventana Medical Systems, Inc) and semi‐quantitatively scored for expression of SOX17, SOX2, keratin 7, keratin 17, and p16. Images were viewed and selected using Image Viewer Software (Ventana MS).

### 2.3 HPV genotyping and detection by ISH

HPV genotyping was performed with the multiplex ligation‐dependent probe amplification assay (MLPA) or the Single tube Multiplex Amplification in Real Time (SMART) kit (PathoFinder).^36^, ^37^ HPV typing was performed on DNA isolated from 4‐µm thick FFPE tissue sections or DNA isolated from cytological samples matching with the tissue obtained after colposcopy and processed for histological examination.

HPV was detected in FFPE tissue sections by means of ISH, using either fluorescence (FISH) or chromogenic detection (CISH), with DNA probes for HPV 16, 18, and 31. For a detailed description of HPV ISH, see Materials and Methods S1. The FISH and CISH signals were classified according to their distribution patterns, typical for the presence of episomal viral copies, viral replication or viral integration into the human genome.^38^

### 2.4 *SOX17* promotor CpG island methylation

DNA was isolated from cells manually dissected from FFPE sections. The areas of dissection were selected on basis of p16 and SOX17 immunostaining, or ISH HPV positivity (see Materials and Methods S1). DNA was bisulfite treated (using the EZ DNA Methylation‐Direct Kit; Zymo Research) and after conversion, the potentially methylated *SOX17* DNA was amplified. Subsequently, specific primers for the methylated and unmethylated CpG island were applied (Table S2).^39^, ^40^

## 3 RESULTS

### 3.1 Expression patterns of SOX17 and SOX2 in normal and metaplastic adult cervical epithelia

**FIGURE 1:**
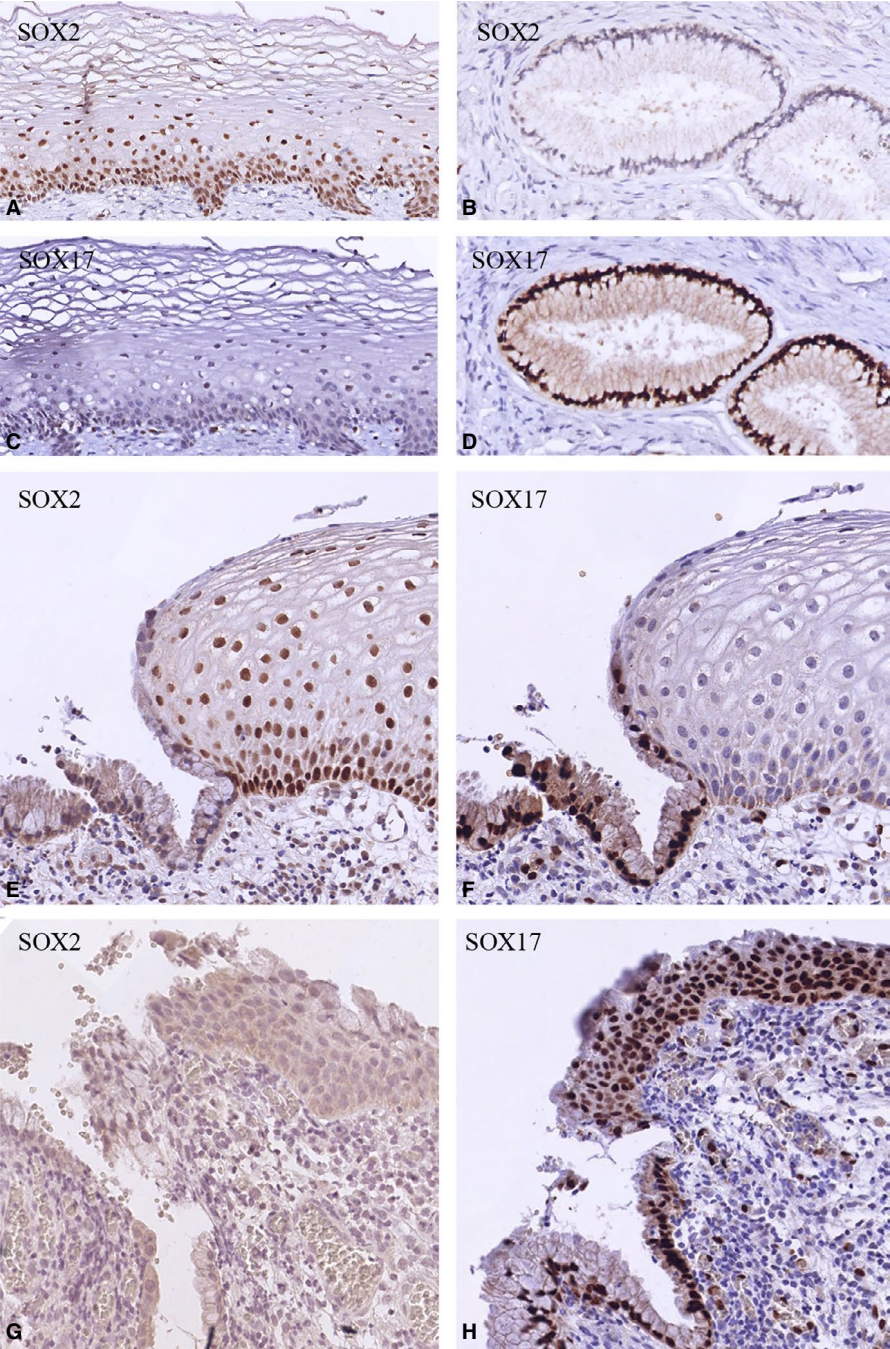
SOX expression patterns in normal cervical epithelia. A‐D, Mutually exclusive SOX2 (A, B) and SOX17 (C, D) immunostaining patterns of normal ectocervical squamous epithelium (A, C) and endocervical glandular epithelium (B, D). This switch in SOX expression is clearly seen at the SqCJ (E, F). SOX2 is negative in immature metaplastic epithelium (G), while this epithelium is positive for SOX17 (H)

Normal adult cervical epithelia, defined as such by their morphology and the fact that they are HPV negative, were analysed in the 12 normal cervices and in 23 cases of cervical preneoplasia. As shown before,^35^ these normal epithelia of the uterine cervix show a mutually exclusive expression pattern for SOX17 and SOX2, with an abrupt transition at the SqCJ between the endocervical epithelium and the squamous type epithelium. The squamous epithelium is characterized by a nuclear staining of SOX2 throughout the epithelial thickness, with exception of the most superficial cells and the strongest immunostaining in the basal cell compartment (Figure 1A,E). In contrast, SOX17 is not found in the ectocervical squamous epithelium (Figure 1C,F), but is extensively expressed in the endocervical columnar epithelium (Figure 1D,F) which is SOX2 negative (Figure 1B,E). New SqCJs are formed within the columnar epithelium in the TZ, typically showing small fields of squamous metaplasia bordering the cervical epithelial invaginations (shown in an overview in Figure S1A,B). In contrast to what is seen in the squamous epithelium of the original SqCJ (OSqCJ), these regions of immature squamous metaplasia can show a strong immunostaining for SOX17 (Figure 1H), but are generally negative for SOX2 (Figure 1G) or can occasionally show a weak cytoplasmic reactivity for this marker (Figure S1C,D).

**FIGURE 2:**
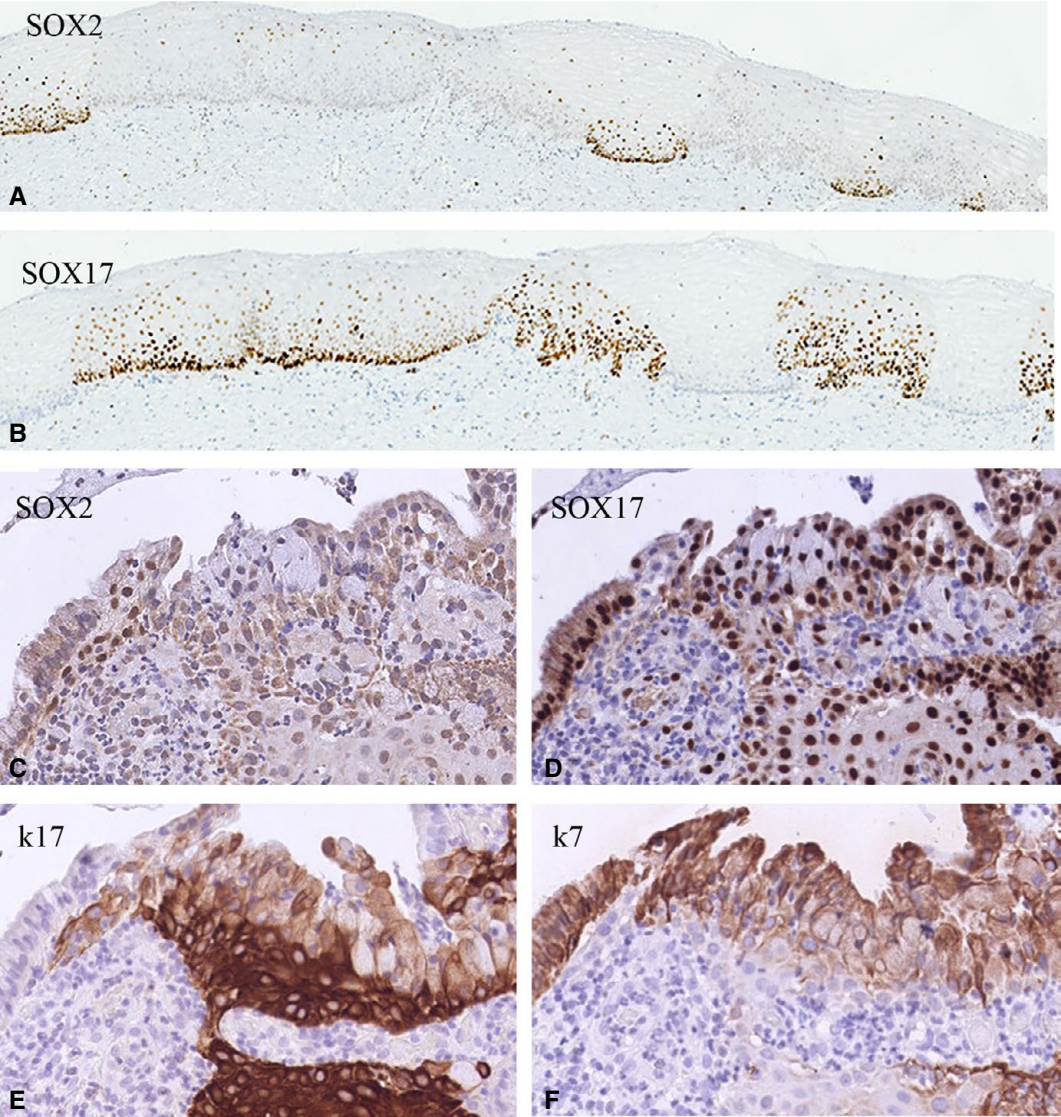
SOX expression patterns in metaplastic cervical epithelia. A and B, SOX2 (A) and SOX17 (B) immunostaining in corresponding areas morphologically classified as mature squamous epithelium, showing alternating and mutually exclusive expression of SOX2 and SOX17. C‐F, SOX2 is negative in immature metaplastic epithelium (C), while this epithelium is positive for SOX17 (D), with the strongest expression in the basal cell layers. Immature metaplastic squamous epithelium is characterized by a strong positivity for keratin 17 mainly in the basal cell layers (E) and a slightly weaker, more superficial positivity for keratin 7 (F)

In such an adult TZ, which is a dynamic area of a few millimeters in length, stretches of SOX2‐positive and SOX17‐negative squamous epithelium can alternate with areas of SOX17‐positive and SOX2‐negative regions (compare Figure 2A,B). Such SOX17‐positive areas were seen in 2 out of 12 (16.7%) normal cases and in 5 out 23 (21.7%) samples of the morphologically normal squamous epithelia adjacent to SIL lesions (see also Figure 2C,D). They were characterized by a strong positive immunostaining for keratin 17 (Figure 2E), a weaker keratin 7 staining (Figure 2F), and absence of HPV, either concluded on basis of absence of p16 or a negative HPV ISH (results not shown). Based on these characteristics, and their squamoid morphology, these SOX17‐positive areas are defined as (remnants of) immature squamous metaplasia.

### 3.2 Expression of SOX17 in reserve cells

**FIGURE 3:**
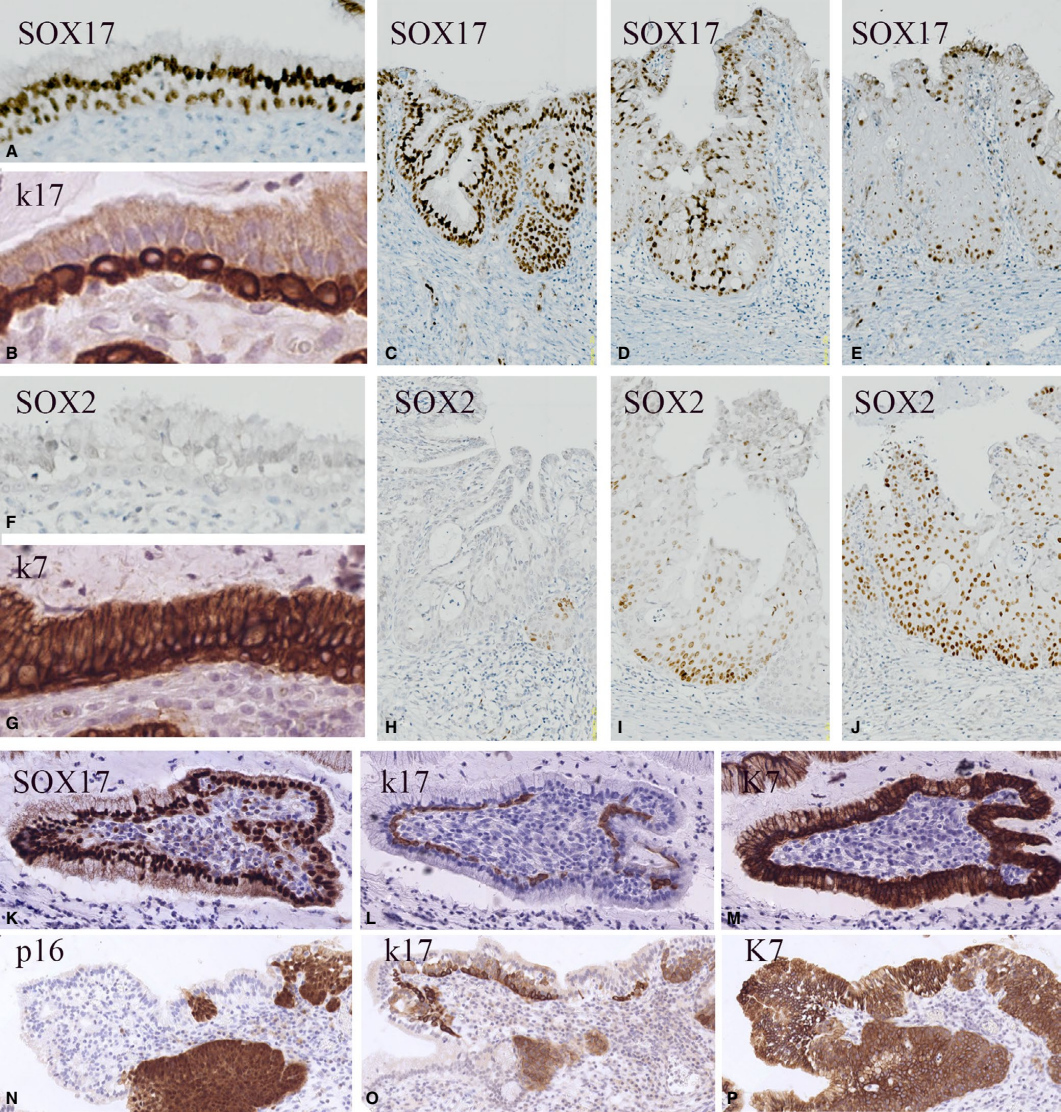
SOX expression patterns in cervical reserve cells. SOX17 immunostaining of reserve cells in normal epithelium found in cases of microglandular hyperplasia (A‐J) and SIL (K‐P). A‐E, SOX17 immunoreactivity in endocervical glandular epithelium and reserve cells (A), microglandular hyperplasia (C), and in early immature metaplasia (D, E). F‐J, Increasing expression of SOX2 is seen upon development of immature metaplasia (F, H‐J). Keratin 17 (B) shows a specific immunostaining reaction in the reserve cell compartment, while keratin 7 (G) additionally also stains the columnar/glandular epithelial cells. K‐P, SOX 17 positivity (K) in keratin 17 (L) and keratin 7 (M)‐positive reserve cells in a case of high‐grade squamous intraepithelial lesions. These stretches of reserve cells may be positioned in close proximity to the HPV‐positive premalignant lesion as shown in (N‐P) after immunostaining for p16 (N), keratin 17 (O), and keratin 7 (P)

Although present in a scattered pattern in most of the cervical biopsies, reserve cells were easily found in the cases with microglandular hyperplasia. In these lesions, a strong SOX17 positivity was noted in both reserve cells and columnar epithelial cells (Figure 3A,C), which were negative for SOX2 (Figure 3F,H). Reserve cells can be identified by a strong immunostaining reaction for keratin 17 and to a lesser extent by keratin 7 positivity (Figure 3B,G). Upon metaplastic transformation, SOX17 and SOX2 show co‐expression in small focal, basal areas (compare Figure 3D,I), which upon further development change into a SOX2‐positive hyperplastic epithelium with occasionally SOX17‐positive superficial cells (Figure 3E,J). Reserve cells found in cases of SIL, as identified by their keratin 17 (Figure 3L) and keratin 7 (Figure 3M) expression pattern, are also SOX17 positive (Figure 3K) and SOX2 negative (result not shown). These stretches of reserve cells can be found adjacent to or in close proximity to the premalignant lesion (Figure 3N‐P).

### 3.3 Expression of SOX17 and SOX2 in squamous (pre)malignant lesions

The expression patterns of SOX17 and SOX2 were semi‐quantitatively assessed in 80 cases of squamous premalignant (SIL; n = 60), including 10 cases of co‐existing SIL/AIS lesions, and malignant lesions (SCC; n = 20) (Table 1). In 53 out of the 60 (88.3%) SIL lesions (both LSIL and HSIL and including the SIL lesions in the co‐existing SIL/AIS), an exclusively SOX2‐positive staining pattern (n = 45; 75%) or dominant expression (n = 8; 13.3%) of SOX2 was seen. In these positive lesions, at least 20% of the cells expressed SOX2 with the strongest staining intensity in the basal cell compartment. Only two cases showed a dominant or exclusive expression of SOX17, while five cases were completely negative for both SOX2 and SOX17. Co‐expression of SOX17 with SOX2 was found in 8 out of 60 cases (13.3%). All 20 SCCs showed an exclusive SOX2 expression in 30%‐100% of the cells. In the co‐existing squamous and glandular (SIL/AIS) premalignant lesions, the squamous component exclusively expressed SOX2 in 7 of the 10 (70%) cases, while most of the AIS regions were positive for SOX17, as described before.^35^

**TABLE 1:** Overview of SOX17 and SOX2 expression patterns in (pre)malignant lesions of the uterine cervix. The different classes of SOX17 and SOX2 combinations are based on staining intensity and percentage of positive cells

Type of tissue			SOX17+ only	SOX2+ only	SOX17+/SOX2+	SOX17−/SOX2−
LSIL	n = 17		1	12	3	1
HSIL	n = 33		0	26	4	3
Co‐existent SIL/AIS	n = 10	SIL	1	7	1	1
		AIS	5	1	3	1
SCC	n = 20		0	20	0	0

Abbreviations: AIS, adenocarcinoma in situ; HSIL, high‐grade SIL; LSIL, low‐grade squamous intraepithelial lesion; SCC, squamous cell carcinoma.

### 3.4 Switches in SOX expression in the TZ of SIL lesions

**FIGURE 4:**
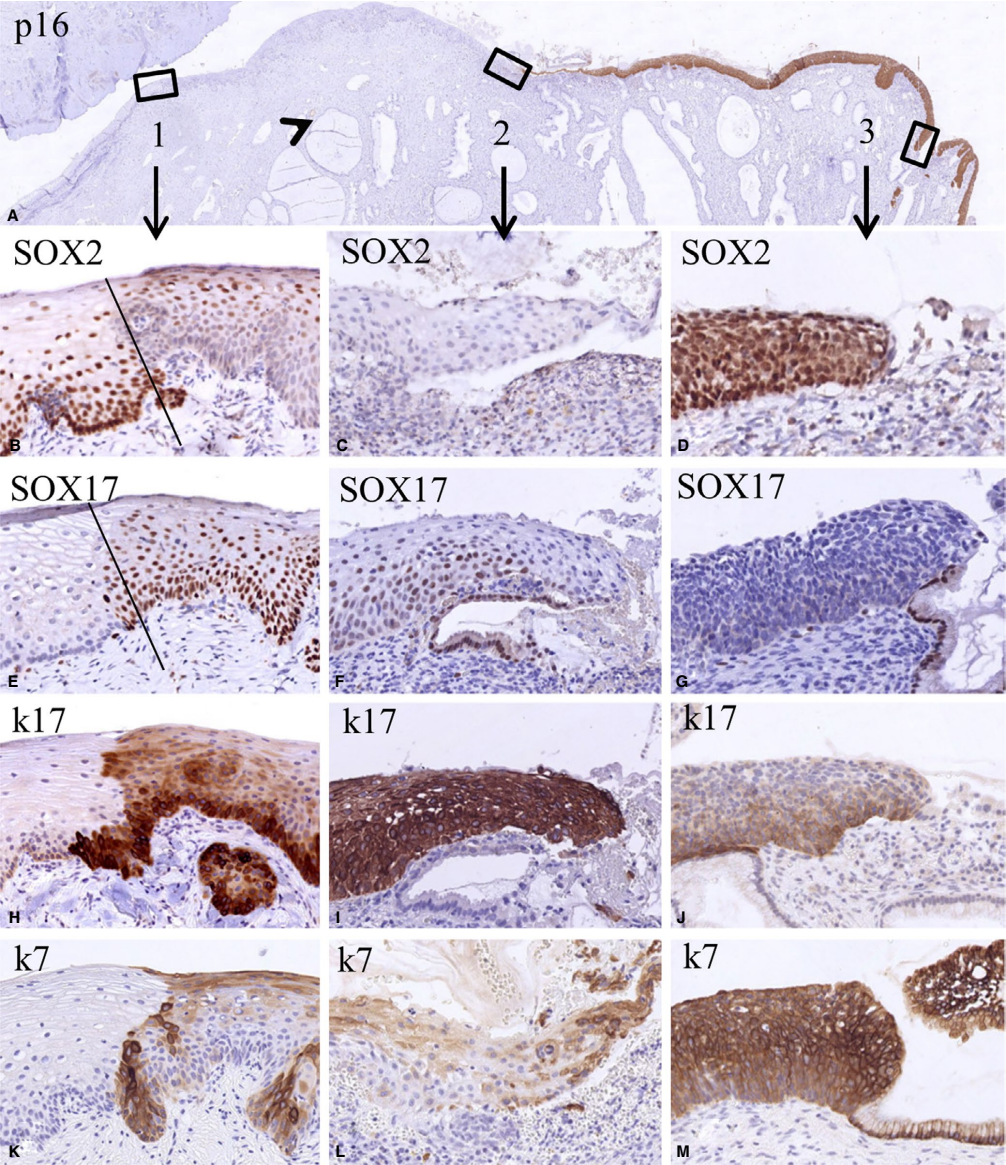
Switches in the expression of SOX2 and SOX17 in the transition of normal epithelium to high‐grade squamous intraepithelial lesions (HSIL). Low magnification of a section stained for p16 showing negative normal epithelia and a positive HSIL lesion (A). The boxes 1‐3 in this figure indicate epithelial transitions within the normal epithelium (box 1: a transition between normal and metaplastic squamous epithelium, possibly the original squamocolumnar junction), a new squamocolumnar junction (box 2), and the transition between HSIL and glandular epithelium (box 3). The fact that glands are seen beneath the squamous epithelium between boxes 1 and 2 (arrowhead) indicates that this region can be regarded as the transformation zone. The higher magnifications of these areas (B‐M) illustrate the mutually exclusive expression of SOX2 and SOX17 in these transitions. This is again evident in the keratin 17‐ and keratin 7‐negative normal ectocervical squamous epithelium in the p16‐negative area (left epithelial area in B, E, H, K). The keratin 17‐ and keratin 7‐positive metaplastic epithelium (right epithelial area in B, E, H, K) shows an extensive SOX17 positivity in the basal and intermediate compartment, and in this case SOX2 positivity in the superficial layers. In the strongly keratin 17‐positive squamous epithelium close to the new SqCJ only SOX17 is expressed, while SOX2 expression is absent (C, F, I, L). This SOX expression pattern is reversed in HSIL as identified by expression of keratins 7 and 17, where SOX2 expression is high and SOX17 expression is absent (D, G, J, M; p16 positive area)

Figure 4 illustrates the typical example of the epithelial trajectory from ectocervix to endocervix including several SqCJs and a HSIL, with the glands underneath the squamous epithelium clearly visible (low magnification overview in Figure 4A). In the p16‐negative normal squamous epithelium (box 1 in Figure 4A), an abrupt change in SOX17/SOX2 expression is seen. In the ectocervical part of the epithelium, the typical SOX2‐positive and SOX17‐negative profile is recognized (Figure4B,E). Cranial from this junction the squamous epithelium becomes basally SOX17 positive and SOX2 negative, but remains superficially partly positive for SOX2 (see Figure 4B,C,F showing a NSqCJ present in box 2). Keratin 17 showed staining of the basal epithelial layers (Figure 4H) or staining throughout the total thickness of the epithelium (Figure 4I), while keratin 7 was only faintly and sporadically positive (Figure 4K,L). The p16‐positive HSIL lesion (shown in box 3 at a SqCJ) was SOX17 negative (Figure 4G), completely SOX2 positive (Figure 4D) and expressed keratin 17 and keratin 7 (Figure 4J,M).

### 3.5 Correlation of switches in SOX expression to HPV infection in SIL

To answer the question whether or not HPV infection results in a switch of SOX expression, we studied the association between the topographic localization of SOX17 and SOX2 expressing epithelia and the viral infection as detected by p16 and ISH, using 23 cases of SIL that could be studied in detail (Table 2). We focused on dysplastic and adjacent or co‐existing metaplastic lesions close to the SqCJ, supposed to be the area at risk for viral infection.

**TABLE 2:** Overview of SOX17 and SOX2 expression as scored on basis of Ventana scans, in relation to *SOX17* methylation status, HPV type, and physical status of the virus in (pre)neoplastic cervical lesions

Patients	IHC		*SOX17* methylation		HPV type	CISH		
	SOX17	SOX2	Normal SqE	Lesions		Episomal	Repl	Int
LSIL								
1	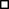	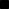	n.a.	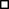	16	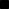		
2	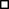	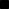	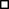	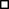	16	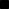	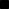	
3	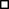	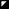	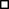	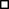	16/31	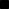 / 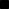	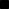 / 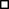	
4	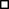	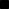	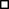	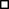	31	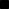		
5	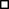	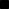	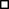	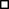	16	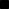	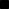	
6	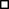	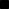	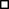	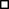	16	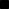	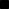	
7	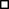	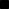	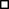	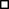	16	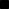	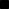	
8	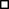	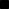	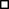	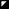	18	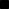		
HSIL								
9	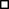	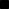	n.a.	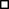	31	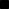		
10	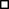	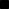	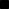	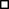	16	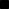		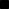
11	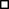	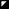	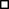	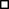	16	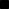		
12	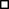	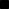	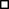	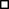	31	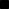	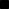	
13	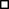	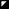	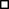	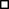	16	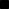		
14	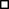	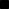	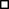	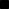	16	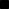		
15	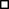	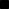	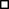	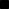	16	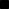	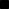	
16	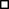	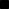	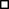	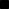	16	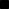		
17	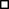	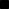	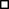	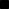	16	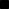		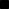
18	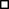	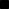	n.a.	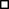 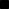	16	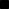	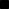	
SIL/AIS								
19	n.a./ 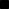	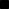 / 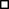	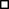	n.a./ 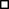	18	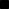 / 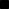	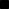 / 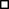	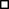 / 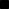
20	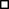 / 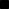	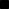 / 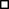	n.a.	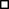 / 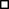	16	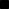 / 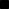	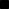 / 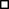	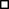 / 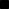
21	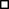 / 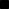	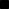 / 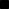	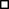	n.a./ 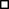	16	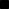 / 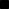		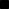 / 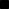
22	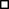 / 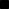	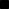 / 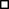	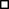	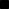 / 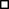	16	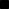 / 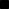		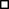 / 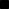
23	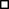 / 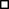	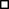 / 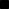	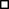	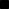 / 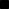	16	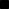 / 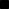		

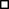
: No immunostaining, no methylation, not present.

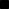
: Positive nuclear immunostaining, methylation, present.

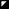
: Faint immunostaining, small fraction of methylated cells.

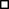
/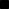: Two concurrent areas analyzed.

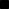
/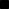: SIL and AIS separately analyzed.

Abbreviations: AIS, adenocarcinoma in situ; HSIL, high‐grade intraepithelial lesion; IHC, immunohistochemical staining; int, integrated HPV; LSIL, low‐grade squamous intraepithelial lesions; n.a.: not available/not suitable for analysis; repl, replicating HPV; SqE, squamous epithelium.

**FIGURE 5:**
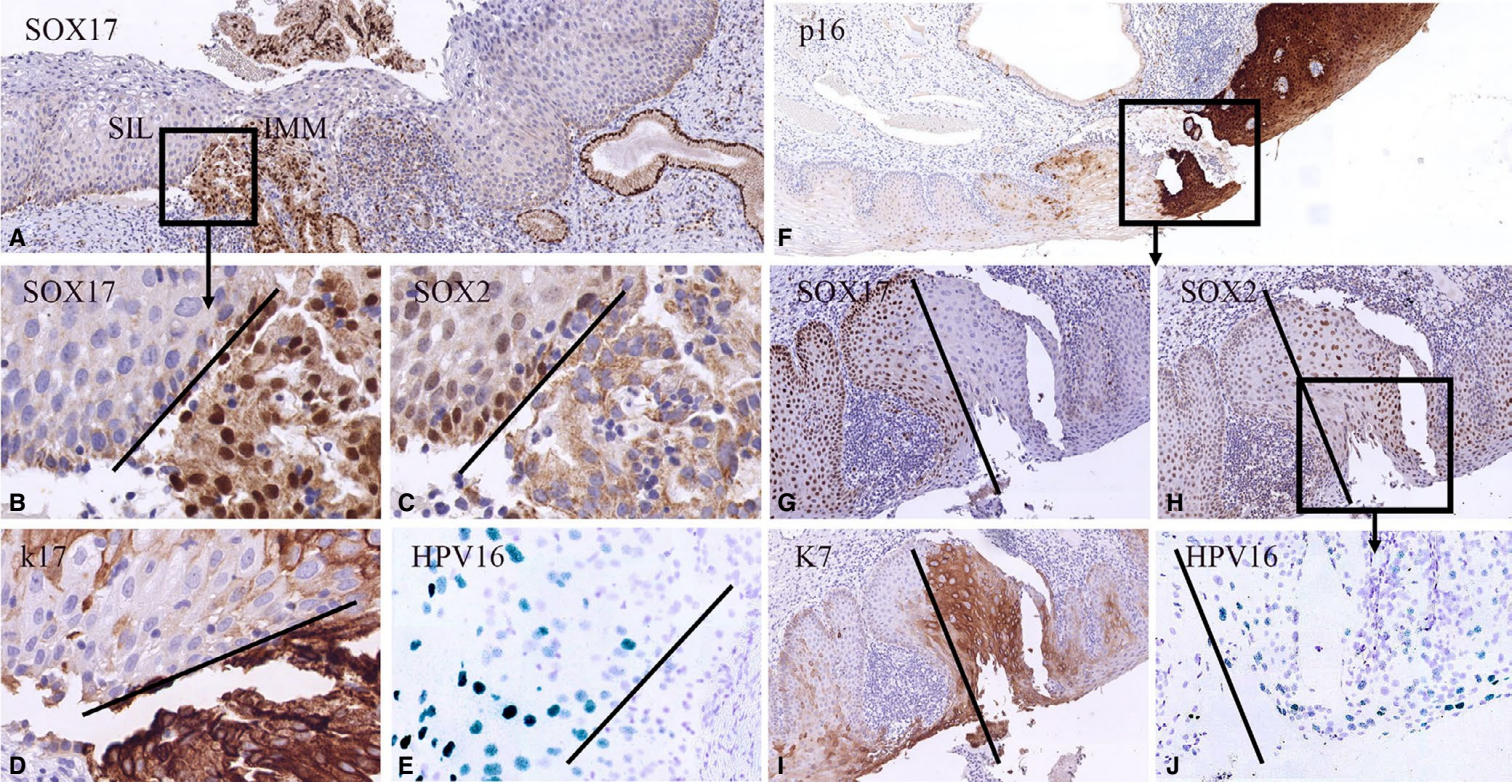
Switches in expression of SOX2 and SOX17 in relation to high risk human papilloma virus infection in SIL. Low magnifications of high‐grade squamous intraepithelial lesions (HSIL) lesions immunostained for SOX17 (A) and p16 (F). These cases show switches in SOX expression in areas containing the keratin 7‐positive HSIL (I) lesions next to keratin 17‐positive (immature) metaplasia (D). The metaplastic areas show SOX17 positivity (B, G) and no expression of SOX2 (C, H). Only the SOX2‐positive regions show HPV 16 positivity as detected by in situ hybridization (CISH; E, J)

Figure 5A‐J show two cases in which the SIL lesion is infected by HPV (as detected by ISH and p16 immunostaining; Figure 5E,F,J) and where the infected regions abut to non‐infected (remnants of) immature metaplasia. At this transition (indicated by the bars in Figure 5B‐J), a switch in SOX expression is seen from a SOX2‐negative and SOX17‐positive staining pattern in metaplasia to a SOX2‐positive and SOX17‐negative pattern in the infected SIL areas (Figure 5B,C,G,H). This correlation between HPV infection and the switch in SOX expression was found in 3 out of 13 cases (23.1%) of SIL where squamous metaplasia (characterized by a strong positive immunostaining for keratin 17 and keratin 7; Figure 5D,I) was found adjacent or in close proximity to SIL (see Table 3).

**TABLE 3:** SOX expression patterns in areas of morphologically normal squamous epithelium (40 areas in 18 SIL cases) either distant from (27 areas) or adjacent/in close proximity (13 areas) to the lesions

Type of tissue		SOX17+ only	SOX2+ only		SOX17+ SOX2+	SOX17− SOX2−
Morphologically normal areas of squamous epithelium including remnants of (immature) metaplasia	n = 27	9	17	0		1
Morphologically normal squamous epithelium adjacent to squamous intraepithelial lesion (SIL) including (remnants) of immature metaplasia	LSIL n = 8	1	7	0		0
	HSIL n = 5^a^	2	3	0		0
SIL	LSIL n = 8	0	7	0		1
		0	10	0		0
	HSIL n = 10					

Abbreviations: HSIL, high‐grade intraepithelial lesion; LSIL, low‐grade squamous intraepithelial lesions.

^a^ From the 10 samples of HSIL shown in Table 2, only 5 showed morphologically normal squamous epithelium adjacent to the HSIL.

In co‐existing SIL/AIS lesions, SOX17 expression was exclusively found in the glandular compartment, while SOX2 can be found in both the glandular and squamous compartments (Table 2) as shown before.^35^

To further corroborate the findings as described above for the three areas in cases of co‐existing metaplastic/dysplastic lesions, we studied the SOX expression patterns in 27 areas of morphologically normal squamous epithelium, including remnants of (immature) metaplasia, distant from the SIL lesions. Table 3 shows that of these 27 regions of apparently normal/metaplastic squamous epithelium 9 (33.3%) were exclusively positive for SOX17, while 17 (63%) of these areas showed an exclusive SOX2 expression pattern.

### 3.6 *SOX17* Promotor CpG island methylation in squamous premalignant lesions

To explain the loss of SOX17 expression during the transition of reserve cells into SIL, the promotor CpG island methylation status of *SOX17* was determined in these lesions. In normal squamous epithelium, which was used as control, the examined CpG island in the promotor region of *SOX17* was methylated in only 1 out of 19 cases (5.3%) (Table 2). Since downregulation of SOX17 protein expression is also seen in the transition of immature metaplasia into SIL, the methylation status of *SOX17* was assessed in these squamous preneoplastic lesions, of which 12 out of 18 cases (66.7%) did not show methylation, while in 6 of the 18 (33.3%) LSIL/HSIL the promotor region was methylated, one case of LSIL and five cases of HSIL. In the co‐existing SIL/AIS lesions, the association between methylation status and protein expression of SOX17 was lacking in SIL, while in AIS the loss of SOX17 expression runs parallel with the methylation of *SOX17* as described before.^35^

## 4 DISCUSSION

The transition zone between glandular and squamous epithelium in several types of mucosa has been described as a high‐risk region for the development of metaplasia and (pre)neoplastic lesions.^5^, ^16^, ^26^, ^41^ The debate about the cells of origin for these lesions is, however, still ongoing.^15^, ^16^, ^42^, ^43^ Neoplasia in the cervix is suggested to occur at particular epithelial sites, where vulnerable cells, such as the reserve cells or cuboidal cells, are found.^14^, ^15^ The underlying study focuses on SOX2 and SOX17 expression during (pre)neoplastic transformation of the uterine cervix. In an earlier study, we found a mutually exclusive expression pattern of these transcription factors at the SqCJs in the normal TZ of the adult cervix.^35^ In the underlying study, we use their expression patterns to explain the processes taking place in the dynamic area of the TZ during the formation of metaplasia and upon HPV infection resulting in SIL.^3^, ^9^ This TZ is situated between the OSqCJ and the NSqCJ, in the formation of which reserve cells play an important role.^4^, ^6^, ^13^, ^44^

**FIGURE 6:**
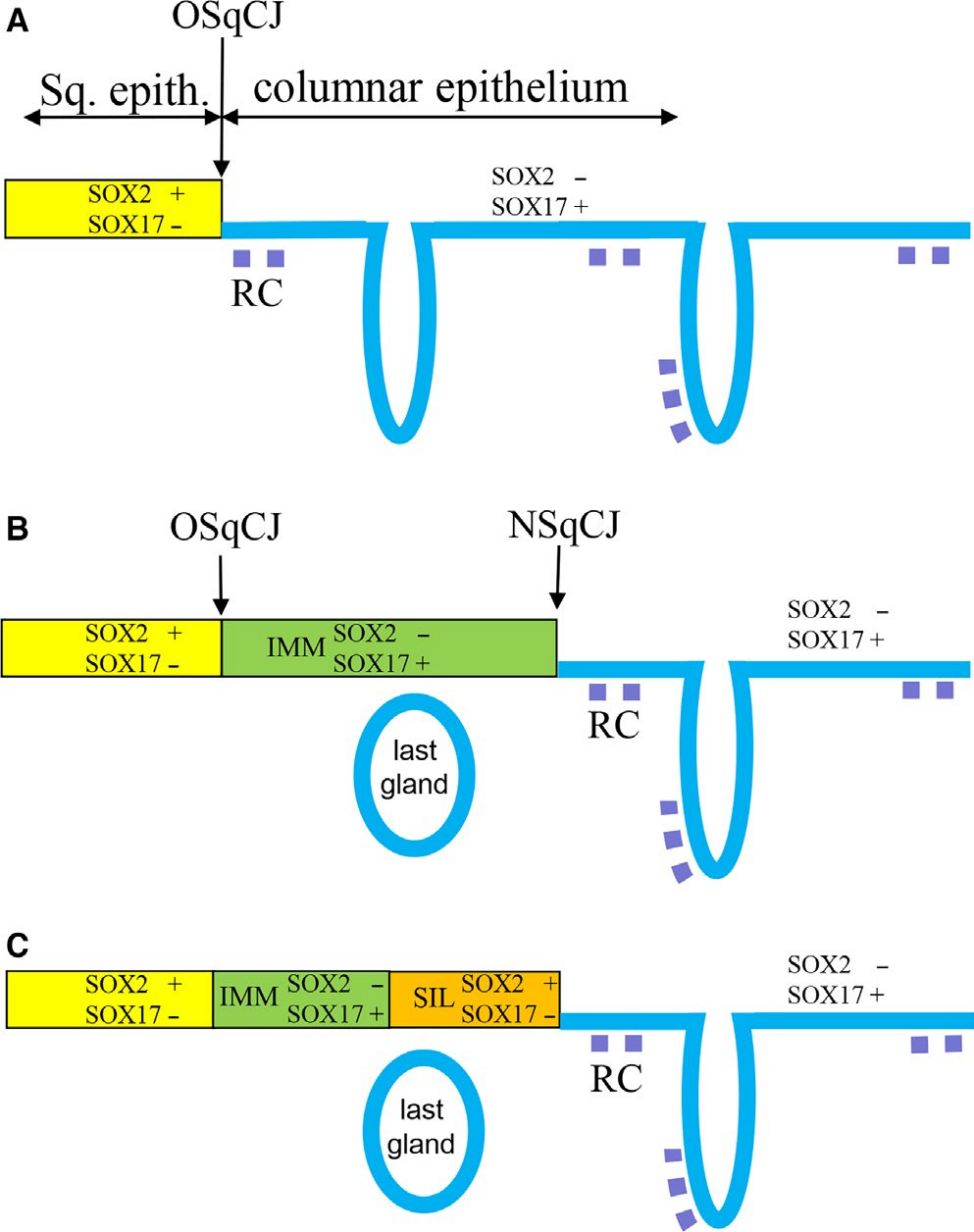
Schematic overview of switches in SOX expression during formation of metaplasia and SIL in the cervical transformation zone. IMM, immature metaplasia; LG, position of the last gland (sometimes seen as Nabothian cyst) beneath the squamous epithelium formed during the metaplastic process; NSqCJ, new squamocolumnar junction at the interface between metaplasia and columnar epithelium; OSqCJ, original squamocolumnar junction at the interface between squamous epithelium and columnar epithelium; RC, reserve cells

Their specific immunostaining patterns distinguish between the SOX2‐positive ectocervical squamous epithelium on the one hand, and the SOX17‐positive endocervical glandular (columnar) epithelium on the other (Figure 6A). Reserve cells could express SOX17 but not SOX2. In the development of metaplasia, reserve cells show a changing SOX‐expression pattern from SOX17 positivity alone, via a complex combination of SOX17 and SOX2 in immature metaplasia (IMM), to exclusive SOX2 expression in areas of mature squamous metaplastic epithelium (indistinguishable from normal squamous epithelium) (Figure 6B). We found that in squamous epithelium with an apparently normal histological appearance, areas can be recognized that exhibit the SOX expression pattern of IMM (SOX17 positive and SOX2 negative), an observation that is supported by the typical keratin 17 positivity and absence p16 in these IMM regions.^6^ The comparable SOX17 expression patterns in reserve cells and in the remnants of IMM support the general opinion that reserve cells are the progenitors for IMM and may therefore provide additional evidence for the reserve cell as the progenitor of cervical (pre)neoplasia.^13^, ^22^, ^43^, ^45^ The reserve cells, in turn, are suggested to originate from either the basal cells of the squamous epithelium^14^, ^46^ or from the stretch of cuboidal cells at the SqCJ recognized by Herfs et al^15^ Although we did not specifically recognize these stretches of cuboidal cells in our tissue samples, it is very likely that these latter cells are also SOX17 positive.

In the literature, three different pathways have been suggested to lead to the development of (pre)neoplastic and finally invasive cervical cancer. The first, well described and accepted pathway suggests that HSIL develops via LSIL and that LSIL originates in the ectocervical epithelium by high risk human papilloma virus (hrHPV) infection of the basal cell compartment in the squamous epithelium.^24^, ^41^, ^47^, ^48^ A second theory states that HSIL is the result of a hrHPV infection of IMM, as concluded from the dual expression of keratin 17 and p16 in atypical squamous lesions with metaplastic features.^6^, ^13^, ^22^, ^43^, ^45^ The third pathway involves hrHPV infection of the specific stretch of endocervical, keratin 7‐positive cuboidal cells at the SqCJ, producing immediately a HSIL without a low‐grade precursor lesion.^4^, ^5^ Our results support the view that IMM, originating from reserve cells is the target for hrHPV and as a result the origin of HSIL, concluded from the fact that we found a switch from SOX17‐positive and SOX2‐negative areas of squamous epithelium (IMM) to SOX17‐negative and SOX2‐positive HSIL which were in direct connection with each other (Figures 5 and 6C). These observations support previous reports that showed HPV positivity by PCR or p16 immunoreactivity in atypical immature metaplasia.^22^, ^23^

It was shown previously that SOX2 expression increased with severity of SIL lesions, but data in the literature are contradictory.^27^, ^28^, ^29^, ^30^, ^31^, ^32^, ^33^, ^43^ In our study, we detected an equally strong nuclear expression of SOX2 in the normal squamous epithelium, as compared to the majority of LSIL, HSIL, and SCC. Although it is difficult to draw conclusions about the differences in SOX2 expression levels based on immunohistochemical procedures, different experimental conditions used (eg, antibody dilution and secondary detection system) could explain these discrepancies.

## 5 CONCLUSION

It is generally accepted that the TZ of the uterine cervix is the target area for hrHPV infection. Several studies hypothesized that in the formation of HSIL HPV first infects a small population of non‐stratified cuboidal cells, directly proximal to the SqQJ.^15^, ^41^, ^49^ In this model, the infected cuboidal cell initially dedifferentiates into reserve cells, which subsequently give rise to the preneoplastic lesion.^41^ Our results on SOX2 and SOX17 expression in the normal and preneoplastic cervix suggest that also existing reserve cells and/or metaplastic cells can be the progenitors for HSIL. In this view, the multifocal patches of remnant columnar epithelium between areas of squamous metaplasia in the endocervix should be taken into account.^6^ In our study, we showed that these NSqCJ, with metaplastic areas and reserve cells beneath the glandular epithelium, are new potential targets for HPV infection.

## CONFLICT OF INTEREST

The authors are responsible for disclosing all financial and personal relationships between themselves and others that might bias their work. There are no potential conflicts.

## AUTHOR CONTRIBUTIONS

Moshi JM: writing; sorting data for normal cervices, SIL lesions; literature search; and interpretation of IHC/FISH/CISH data; Hoogduin KJ: selections of (pre)neoplasia; analysis of IHC staining patterns for SOX2, SOX17; and conceptual discussions; Ummelen M: technical performance of IHC staining p16, FISH and CISH, and imaging; Henfling MER: technical performance of methylation, IHC staining of part of sections for SOX2 and SOX17, and imaging; van Engeland M: Dept. Pathology (Pathobiology of Cancer, in particular the role of Epigenetics), responsible for KW, conceptual discussions; Wouters KAD: design primers and methylation assay of SOX17; Stoop H: IHC staining of part of sections for SOX2 and SOX17; Demers I: technical performance of SOX17 IHC and HPV ISH; Looijenga LHJ: as head of Dept. Experimental Patho‐Oncology final responsibility for SH, responsible for SOX staining, conceptual discussions; Ramaekers FCS: as head of the Dept. Molecular Cell Biology final responsibility for team AH, MU, and MH, conceptual discussions and writing; Hopman AHN: conceptual discussions, writing; evaluation of all IHC reactions, methylation data, FISH/CISH results; and imaging, supervising JMM, communication within team. All persons read the manuscript and wrote part for which they were responsible (for technical performance: see above).

## Supporting information

Fig S1

Supplementary Material

## Data Availability

The data that support the findings of this study are available from the corresponding author upon reasonable request.
