# Supplementary figures and images for: Switches of SOX17 and SOX2 expression in the development of squamous metaplasia and squamous intraepithelial lesions of the uterine cervix
Source: Cancer Med. 2020 Jul 9;9(17):6330–43. doi: 10.1002/cam4.3201 (PMC7476841; doi:10.1002/cam4.3201)

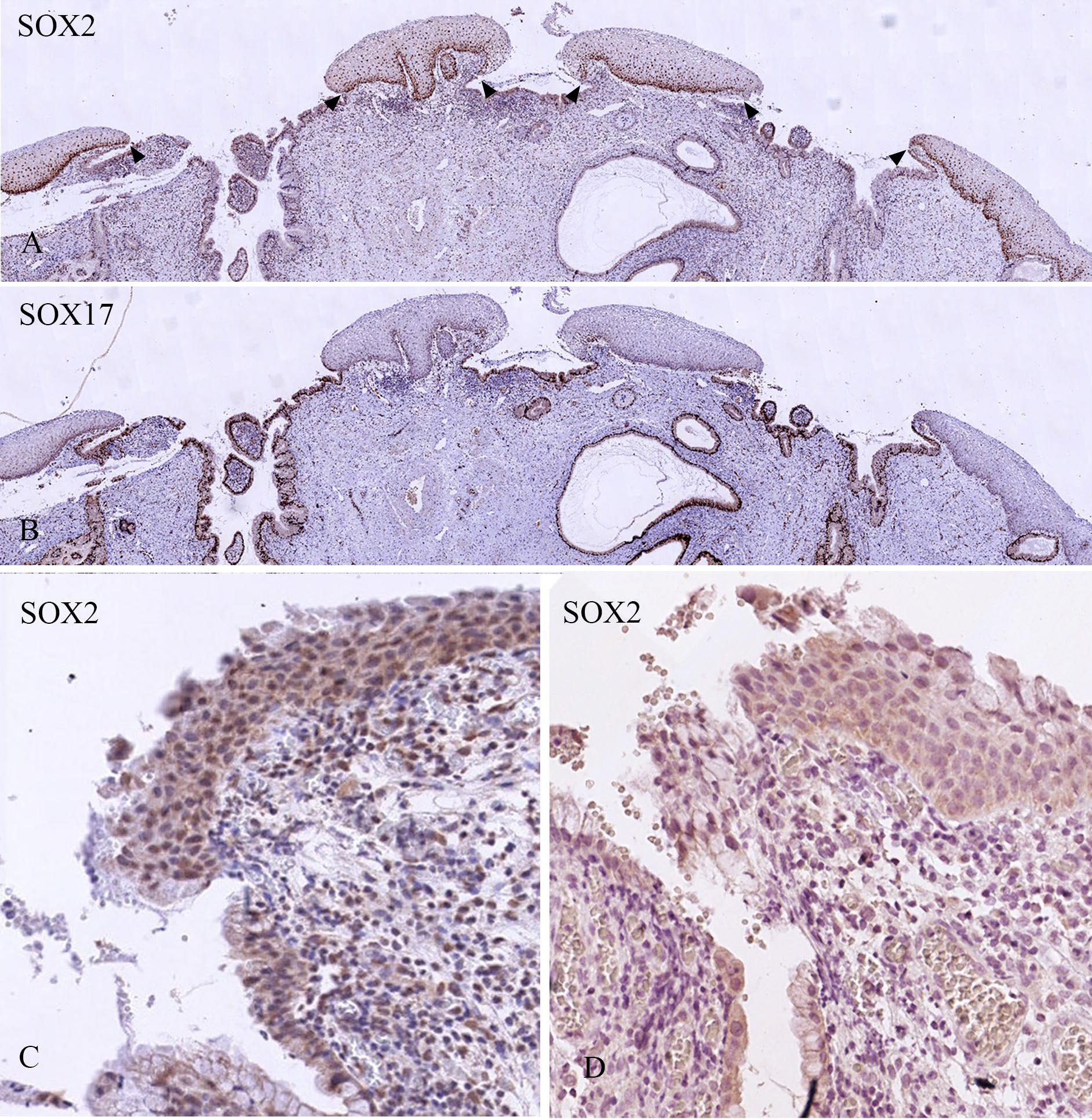

Supplement: Supplementary file 1 — Fig S1 [file CAM4-9-6330-s001.tif]
