# Supplementary material for: Switches of SOX17 and SOX2 expression in the development of squamous metaplasia and squamous intraepithelial lesions of the uterine cervix
Source: Cancer Med. 2020 Jul 9;9(17):6330–43. doi: 10.1002/cam4.3201 (PMC7476841; doi:10.1002/cam4.3201)
Supplement: Supplementary file 2 — Supplementary Material [file CAM4-9-6330-s002.docx]

**Supporting Information.**

**Supplementary Materials and Methods:**

Immunohistochemistry

After standard deparaffinization in xylene and blocking of endogenous peroxidase activity by 0.3% peroxide in methanol, the 4 µm thick tissue sections were boiled and incubated at room temperature to cool down in 10 mM Tris-EDTA buffer pH 9.0 for 20 min in a microwave oven for antigen retrieval. The antigens were detected with the primary antibodies, secondary antibodies and enhancement procedures as listed in Supplementary Table1, stained with diaminobenzidine.HCl (DAB) and finally counterstained with hematoxylin.

It is important to note that we used a dilution of the primary antibody for SOX2 in combination with a sensitive secondary antibody to obtain a positive nuclear staining throughout the epithelium. This could result in some cytoplasmic background staining in the endocervix.

**Supplementary Table S1: Antibody characteristics and optimized immunohistochemical detection methods.**

| **Antigen** | **Primary Antibody** | **Dilution** | **Secondary antibody** | **Enhancement/Detection** |
| --- | --- | --- | --- | --- |
| SOX2 | pAb Goat IgG  AF2018  R&D Systems, Abbingdon,UK | 1:100 in PBST/1% BSA,  1 hr RT | Biotinylated Horse anti Goat IgG, Vector Laboratories, Burlingame, CA, USA,  BA-9500  1:200 in PBST/1% BSA; 30 min RT | ABC, Vectastain Elite ABC Kit, Vector Laboratories, Burlingham, CA, USA,  30 min RT  DAB reaction |
| SOX2 | mAb Rabbit IgG  clone EPR3131  Ab92494  Abcam  Cambridge UK | 1:100 in  PBST/45 min, 37^0^C  5% NGS | Biotinylated Goat anti Rabbit IgG^(6)^, BA-1000  1:200 in PBST/5% NGS  30 min, 37^0^C | ABC, Vectastain Elite ABC Kit, Vector Laboratories, Burlingham, CA, USA,  30 min RT  DAB reaction |
| SOX17 | pAb Goat IgG  GT15094  Neuromics, Edina, MN, USA | 1:2500 in PBST/1% BSA,  1 hr RT | Biotinylated Horse anti Goat IgG, Vector Laboratories,  BA-9500  1:200 in PBST/1% BSA; 30 min RT | ABC  30 min RT  DAB reaction |
| p16 | mAb Mouse IgG2a  Clone E6H4  CINtec, MTM Laboratories AG, Heidelberg, Germany | 1:50 in PBST/1% BSA,  1 hr RT | Biotinylated Horse anti Mouse IgG, Vector Laboratories,  BA-2001  1:200 in PBST/1% BSA; 30 min RT | ABC  30 min RT  DAB reaction |
| Keratin 7 | mAb Mouse  IgG1  Clone OVTL12/30  MUB0316P  Nordic-MUbio, Susteren, The Netherlands | 1:100 in PBST/1%  BSA  45 min, 37^0^C | Biotinylated Horse anti Mouse IgG^(6)^, BA-2001  1:200 in PBST/1% BSA  30 min, 37^0^C | ABC  30 min RT  DAB reaction |
| Keratin 17 | mAb Mouse IgG2b  Clone E3  MUB0325P  Nordic-MUbio, Susteren, The Netherlands | 1:200 in PBST/1% BSA,  1 hr RT | Poly-HRP Goat anti Mouse/Rabbit IgG, Immunologic, Duiven, The Netherlands,  Undiluted  30 min 37°C | None  DAB reaction |

Abbreviations: mAb, monoclonal antibody; pAb, polyclonal antibody; HRP, horseradish peroxidase; RT, room temperature; PBST, phosphate buffered saline + 0.1% Tween-20 (Janssen Chimica, Beerse, Belgium); BSA, bovine serum albumin; DAB, diaminobenzidine; ABC, Avidin-biotin complex with biotinylated HRP; NGS, normal goat serum.

*SOX17* methylation analysis

DNA isolation and bisulfite conversion: The 4 µm thick FFPE tissue sections were deparaffinized in xylol, washed with 100% ethanol and subsequently air dried. Based on hematoxylin and eosin (H&E) staining or specific immunohistochemical staining patterns, cells from the lesions were manually dissected using the edge of an 18x18 mm coverslip (Menzel-Gläser, Braunschweig, Germany). From every sample normal squamous epithelial cells were dissected as controls. The tissue samples were transfered to 26 µl digestion buffer containing Proteinase K (following the instruction of the EZ DNA Methylation-Direct Kit; Zymo Research, Irvine, CA, USA) and incubated for 4 hours at 50°C. Bisulfite conversion was performed by adding 20 µl of digested sample to 130 µl of the C to T (CT) conversion reagent. After incubation for 8 minutes at 98°C and 3.5 hours at 64°C in a thermal cycler, the samples were transferred to a Zymo-Spin IC column, containing binding buffer. After centrifugation and washing of the samples, desulphonation buffer was added to the columns. Following centrifugation and two washing steps, the DNA was eluted in 10 µl of elution buffer.

Methylation specific PCR: Methylation specific primer (MSP) design and MSP analysis on bisulfite-treated DNA was performed as described previously.[^40^](#_ENREF_40) Since only small regions from the FFPE sections were dissected and the DNA yield was expected to be low, the DNA was first amplified with flanking PCR primers, serving as a template for MSP analysis with the specified primers. All PCRs were performed with controls for both methylated DNA (normal human placenta DNA treated in vitro with Sssl methyltransferase (New England Biolabs, Ipswich, MA, USA)) and unmethylated DNA (Epitect unmethylated DNA, Qiagen, Venlo, The Netherlands). The *SOX 17* methylation-specific PCR (MSP) primers are located at chromosome 8 (BLAST and BiSearch genomic region 54458315-54458421) and shown in Supplementary Table 2.

DNA from HeLa cells, which shows bands for methylated and unmethylated DNA was incorporated as a control sample, as well as water controls. Flanking PCRs were performed in a volume of 25 µl containing 200 nM of each primer, 312.5 nM dNTPs (GE Healthcare Europe GmbH, Eindhoven, The Netherlands), 0.5 U Immolase DNA polymerase (Bioline, London, UK) and 4 µl of bisulfite treated DNA. Amplification was performed in a Thermocycler UNO II (Biometra GmbH, Göttingen, Germany), starting with an initial denaturation step of 3 min at 95°C, followed by 35 cycles of 95°C, 56°C and 72°C for 30 sec each, with a final extension step of 4 min at 72°C. A second PCR with primers specific for methylated or unmethylated *SOX17* (see Supplementary Table 2) was performed in a volume of 25 µl containing 400 nM of each primer, 62.5 nM dNTPs, 0.5 U of Immolase DNA polymerase and 4 µl of diluted amplified DNA resulting from the flanking PCR. PCR conditions were as follows: 10 min at 95°C, followed by 35 cycles of 95°C, 56°C and 72°C for 30 sec each, with a final extension step of 4 min at 72°C. PCR reaction products were resolved on 2% agarose gels with Gel Star nucleic acid gel stain (Cambrex Bio Science Rockland, Inc, Rockland, ME, USA) and visualized under UV light. Bands with approximately equal intensity for methylated and unmethylated DNA were scored positive. Faint methylated bands were analyzed again and if persistent considered negative for methylation.[^41^](#_ENREF_41)

In Situ Hybridization

Fluorescence (FISH) or chromogenic (CISH) in situ hybridization: The probes for HPV16, HPV18, HPV31 (PanPath, Uden, The Netherlands) and HPV45 (Dr. E. de Villiers, Deutsches Krebsforschungs Zentrum, Heidelberg, Germany) were labeled with biotin by standard nick translation and used at a concentration of 1 ng/µl in a solution containing 50% formamide, 2 × SSC, 10% dextran sulphate and 50 × excess of carrier DNA (salmon sperm DNA; Sigma, Steinheim, Germany) ISH was performed on 4 µm thick FFPE tissue sections fixed onto Superfrost Plus Microscope Slides (Thermo Fisher Scientific). In brief, to improve adhesion to the slides during the ISH procedure, tissue sections were first heated for 15 min at 80°C. Following heating, the sections were deparaffinized in xylol, hydrated and microwaved for 10 min at 100°C in a 10 mM Na-citrate pH 6.0 buffer, and incubated at room temperature for 20 min to cool down. Subsequently, the sections were washed in demineralized water, rinsed in 10 mM HCl and digested with 2.0 mg/ml pepsin (601 units/mg: porcine gastric mucosa, Sigma, Steinheim, Germany) in 10 mM HCl. Thereafter, the slides were washed once in 10 mM HCl, and once in PBS and post-fixed in 1% formaldehyde in PBS for 5 min at room temperature. After the post-fixation, the slides were washed with PBS, demineralized water and dehydrated in an ascending alcohol series. Subsequently, the HPV probe was applied under a coverslip, simultaneously denatured for 10 min at 80°C and hybridized overnight at 37°C. After hybridization, the slides were washed for 5 min at 42°C in a solution containing 2 × SSC, 0.05% Tween-20 (Janssen Chimica, Beerse, Belgium) and subsequently washed twice for 5 min at 61°C in 0.1 × SSC.

For FISH the hybridized probe was detected with a triple layer detection method, consisting of FITC-conjugated avidin (1:500 dilution; Vector Laboratories, CA, USA), biotinylated goat anti-avidin (1:100 dilution; Vector Laboratories) and finally FITC-conjugated avidin, each for 15 min at RT. The slides were then washed in 4x SSC containing 0.05% Tween-20, dehydrated in an ascending ethanol series and mounted in Vectashield (Vector Laboratories), containing 4',6-diamidino-2-phenylindole (DAPI; Sigma: 0.5 µg/ml). Images were recorded with the Metasystems Image Pro System (black and white CCD camera; Sandhausen, Germany) mounted on top of a Leica DM-RE fluorescence microscope, equipped with FITC and DAPI single band pass filters for single color analysis. Images were obtained using an automatic integration time, allowing semiquantitative evaluation, and using the full dynamic range of the camera without signal intensity saturation.

For CISH, the hybridized probe was detected in a triple layer detection method with peroxidase-conjugated avidin (1: 100 dilution; Vector Laboratories, CA, USA), biotinylated goat anti-avidin (1:100 dilution: Vector Laboratories) and finally peroxidase-conjugated avidin. The enzymatic reaction was performed using Vina Green according to the instructions of the supplier (Vina Green Chromogen Kit, BIOCARE Medical, CA, USA). Finally, the slides were washed in milliQ, counterstained with hematoxylin, immediately dehydrated and embedded in Entellan new, (Merck, Darmstadt,Germany). Images were recorded with the Nikon Eclipse E800 (mounted with a color camera) and Nikon ACT-1 software. HPV physical status as concluded from the ISH patterns was classified as episomal or integrated, and with or without a typical replication pattern.[^39^](#_ENREF_39)

**Supplementary Table S2: *SOX17* Primers Design**.

| **Primer** | **Sense primer (5’→3’) Antisense primer (5’→3’)** | **Annealing temp. (°C)** | **Number of PCR cycles** |
| --- | --- | --- | --- |
| Flank | TGTGTAGGTTTGGATTTTGTTG AACCRAACCAAAAACRAATCC | 56°C | 35 |
| U | ATTTTGTTGTGTTAGTTGTTTGTGTTT ACAAATCCCATATCCAACAACCA | 60°C | 35 |
| M | TTGCGTTAGTCGTTTGCGTTC TCCCGTATCCGACGACCG | 60°C | 35 |
